# The psychosocial wellbeing of orphans: The case of early school leavers in socially depressed environment in Mpumalanga Province, South Africa

**DOI:** 10.1371/journal.pone.0229487

**Published:** 2020-02-26

**Authors:** Busisiwe Ntuli, Mathildah Mokgatle, Sphiwe Madiba

**Affiliations:** Department of Public Health, Sefako Makgatho Health Sciences University, Pretoria, South Africa; King Fahd University of Petroleum & Minerals, SAUDI ARABIA

## Abstract

**Background:**

The emergence of a large population of orphaned youth in sub-Saharan Africa is due to the natural maturity of orphaned children. Research indicates that orphaned youth face more negative psychosocial challenges than their younger counterparts do. Furthermore, these challenges are intensified for early school leavers. This paper describes how experiencing maternal death affects the psychosocial wellbeing of orphaned youth who left school before completing high school.

**Methods:**

An exploratory qualitative study was undertaken among purposively sampled orphaned youth using in-depth interviews with open-ended questions. Fifty participants were recruited through social workers, community based organisations, and tribal authorities in a rural local municipality of Mpumalanga Province, South Africa. All data analyses were performed using NVivo10, following an inductive thematic approach.

**Results:**

The narratives with the participants revealed that they live in a socially depressed environment and are subjected to extreme poverty characterised by frequent hunger. Furthermore, they do not enjoy family support and when they live with their extended families, they experience ill treatment and unsympathetic gestures. The death of their mothers has made a negative psychological impact on their psychosocial wellbeing, resulting in the development of internalising depressive symptoms. They suffer from emotional distress and prolonged bereavement characterised by perpetual yearning for the mother and, they resort to silence as a coping strategy. The study established that they were forced to leave school early for a variety of reasons. However, leaving school early became a major stressor and contributed to their negative psychosocial wellbeing.

**Conclusion:**

Maternal death has a negative impact on the psychosocial wellbeing of the participants even after they have crossed the 18 years threshold of orphan hood. Yearning for their mothers negatively affected their ability to develop coping strategies, which led to isolation, sadness, hopelessness, lack of peace, and fear of an uncertain future. The lack of routine screening for mental health in schools and other settings in South Africa increases their vulnerability to undiagnosed depression. The school health services should develop interventions for mental health screening in schools. For early school leavers, relevant policies should consider the continuation of support through NGOs and community networks.

## Introduction

Sub-Saharan Africa (SSA) is experiencing a high number of orphans due to the prevalence of HIV/AIDS [1]. The long-term outcome of the HIV/AIDS crisis is the emergence of a large population of orphaned youth or young adults due to the natural maturing or aging process of orphaned children [2]. The United Nations defines youth as persons between the ages of 15 and 24 years [3]. In 2011, UNICEF’s estimations indicated that 17.3 million children younger than 18 years of age lost one or both parents to HIV/AIDS related illnesses, and about 90% of these orphans were living in SSA [3]. In 2018, there were approximately 3.8 million orphans because of HIV/AIDS related deaths in South Africa [4].

Orphans have always relied on the extended family as a traditional support system for their care, but this has changed because household structures have been drastically altered by the HIV/AIDS epidemic. The clan network has collapsed and the kin support system has gradually diminished [5–8]. Consequently, communities and families find themselves ill-equipped to cope with the ever-increasing number of orphans in countries experiencing high prevalence of HIV/AIDS, high levels of poverty, and HIV/AIDS related stigma [8, 9]. Due to limited resources, poverty, and the high number of HIV/AIDS related deaths, the extended family is unable to cope with this role and finds it difficult to integrate the orphans into their own families as this may deplete their own resources [8, 10]. Furthermore, UNICEF maintains that the successive loss of multiple family members to HIV/AIDS has led to the gradual erosion extended family safety net of the children [5].

The collapse of the network and kin support system has given rise to the existence of child and youth headed households [8], a common and integral part of South Africa society [7]. Typically, in a child or youth headed household, the main caregiver is younger than 18 years of age. Usually this is the older sibling who takes on the responsibilities commonly assumed by parents to take care of younger siblings after the death of the mother or both parents. van der Mark [7] speaks of the ‘sibling headed households’ concept and argues that some families are being headed by siblings who have turned 18 years and are officially no longer regarded as child headed households anymore. Other researchers refer to this concept as a ‘youth headed household [11]. Nevertheless, in many parts of SSA, orphans continue being cared for by elderly female caregivers, particularly maternal grandparents [12, 13]. The presence of extended families enables orphans to receive support and withstand severe psychological stress [14].

The literature indicates that orphans experience emotional and psychological distress following the deaths of their parents, which leads to their poverty, their exploitation in the homes of their relatives, and their loss of educational opportunities [15]. The emerging evidence suggests that older orphans are at risk of poorer psychosocial outcomes [11], as the negative mental health outcomes amongst orphans are maintained and worsen into later adolescence [16]. Furthermore, older orphans have higher risk of school dropout which is heightened by lack of kin support [17–19]. Different reasons for the high risk of school dropout are documented. For example, being a maternal orphan renders a child nutritionally vulnerable and susceptible to school dropout [20], and when orphans are in child or youth headed households they are more likely to suffer negative impacts on their educational needs [7, 21, 22]. Older siblings in particular drop out of school to find employment in order to take care of the younger siblings [8, 13, 23, 24].

The death of a parent gives rise to emotional distress. The orphans are susceptible to long-term psychological problems including depression, anger, anxiety, and feelings of sadness, and are inclined to withdraw and self-isolate. These psychological problems are brought about by their failure to deal with their sense of loss [17, 25–27]. Those living in child or youth headed households experience hidden grief manifested as a prolonged bereavement [28]. One other key challenge and source of distress to orphans heading households is to adjust to the role of taking care of their siblings unprepared and with no kin support [29, 30].

Much focus has been given to the challenges of orphans in child or youth headed households depicting them as vulnerable due to HIV/AIDS [25]. Lethale and Pillay [31] argue that literature tends to portray a bleak picture of the experiences of orphans in these households even though evidence suggests that some display strong resilience in their academic and personal lives despite the odds. Resilience is defined as the positive adjustment in the context of significant adversity, or risk factors that are known to be associated with negative outcomes [32]. In the context of orphans, resilience is a positive adaptation or a useful weapon for survival following parental death [33]. There’s evidence that, although orphans in child or youth headed households experience lack of food security, poverty, and strained extended family relations, they are resilient and become independent agents and decision makers in their own right [34, 35].

Previous work on orphans in South Africa has focussed on describing their material needs and living arrangements [36]. Literature on the well-being of orphaned youth, in particular, is scanty. Moreover, the emergence of a large population of orphaned youth or young adults due to the natural maturing process of orphaned children [2] calls for an urgent need to understand their unique experiences. Notwithstanding that orphaned youth face more negative challenges than their younger counterparts do because they have been stripped of eligibility for the interventions available for younger orphans [11]. For example, in South Africa, the social grant system is withdrawn from HIV orphans when they reach the threshold of 18 years despite their precarious circumstances such as being the head of their households. Furthermore, research has established a high risk of school dropout among this population [17–19].

This paper describes how experiencing maternal death affects the psychosocial wellbeing of orphans who dropped out of school after the death of their mothers as informed by their own narratives. There is a need to understand how orphan-hood impacts on their psychosocial wellbeing in order to develop context based interventions to address their wellbeing. This is crucial in a country with 3.8 million HIV/AIDS orphans, and child and youth headed households [8] being a common and integral part of society [7].

## Methods

### Study design

This manuscript is derived from a concurrent exploratory mixed-method community-based doctoral study of the lead author, conducted in the Chris Hani local municipality of Mpumalanga Province. The purpose of the main study was to explore the school attainment of orphans, in order to inform the development of an intervention to improve the school outcomes of orphans in secondary schools. The reason for adopting a mixed method (MM) approach, as explained by Ivankova and Greer [37], was to provide more comprehensive answers to the questions posed by the researcher. The qualitative approach allowed for deeper understanding of the meanings associated with subjective well-being from the perspectives of participants. We adopted the definition of Casas [38] where wellbeing is broadly defined as encompassing dynamic processes and the degree to which an individual is fully functioning in society. Goodman [39] recommend that studies aimed at determining subjective well-being include both quantitative and qualitative methodologies.

### Setting and population

Thembisile Hani local municipality, the study setting, is one of the six sub-districts of Nkangala District in Mpumalanga Province, South Africa. The municipality comprises rural villages with poor infrastructure such as poor roads and sanitation. The unemployment rate is high at 37% compared to the national average of 32% with youth unemployment sitting at 50% [40].

The study population consisted of orphans in school and those who dropped out of school prematurely. UNICEF defines an orphan as a child under 18 years of age who has lost one or both parents to any cause of death [41]. Mpumalanga Province is among the provinces that have high rates of orphans and bear a large burden of care for orphans. The province recorded 16.5% of children as orphans who have lost a mother, a father or both parents. The target population for the study was maternal orphans and this category make up 3.5% of the total number of orphans in the province [4]. We used the provincial data because there are no specific statistics for the study setting. As such, the researches recruited the participants with the help of social workers, community based organisations, tribal authorities, community workers, and research assistants with a substantial understanding of the study setting and the context thereof. The participants were recruited if they were; 1) maternal orphans, 2) 18 years and older, and 3) had dropped out of school at the time of the interview. A maternal orphan is defined as a child under 18 years of age who has lost a mother to any cause of death [41]. The Department of Basic Education in South Africa defines dropout as leaving school before completing a given grade in a given school year [42]. The operational definition for school dropout in this study is “Leaving school early before completing high school (12^th^ Grade)”. In South Africa, approximately 6.5% of learners drop out in 9th grade, and about 11% drop out in 10^th^ and 11^th^ [43].

### Data collection and measures

The first author (BN) and a team of research assistants (research team) collected data over a period of nine months between March and November 2016. At the time of data collection, the research assistants already possessed skills in carrying out interviews. They obtained their skills through training received from the Department of Public Health, where the lead author had registered for her doctoral study. However, prior to commencing with data collection, the authors were engaged in the training of the research assistants to understand the study objectives, the data collection tool and the process of data collection. In addition, the inclusion criteria for the prospective participants was also explained to them.

The research team collected data using in-depth interviews (IDIs) with a semi-structured interview schedule with open-ended questions. The interview schedule was translated into two local languages, IsiZulu and Sepedi, which were used during the interviews. The researcher team asked the participants about their schooling experiences before they dropped out of school. The questions focused on; 1) challenges to attend school on a regular basis, 2) support received from the school and community to attend classes, 3) reasons for absenteeism, 4) suggestions on how the school can assist orphans to attend school regularly, 5) what would have made it possible to complete school, and 6) their reasons for dropping out of school.

The research team conducted 50 in-depth interviews in settings including the homes of the participants, the offices of the NGOs, and offices of the tribal authorities. The team conducted all the interviews in private after obtaining informed consent from the participants and consent to record the interviews. Each interview lasted for about 45 minutes. Data saturation guided the collection of data, and thus the interviews ended when no new information emerged from subsequent interviews. Each participant received refreshment after the interview and a food parcel to take home.

### Data analysis

A thematic approach guided the data analysis [44]. All the authors (BN, MM and SM) were involved in the analysis to reduce bias and enhance the credibility of the findings. The research assistant involved in the data collection transcribed the audio files verbatim to so that the findings reflected the meanings as described by the participants [45]. The research team later translated the transcripts into English, and proofread and formatted them for analysis. A few transcripts were repeatedly read by individual authors to familiarise themselves with the depth and the breadth of the data [46]. In order to identify codes and themes, the three authors independently coded a few transcripts and then compared the application of coding for inter coder reliability.

This was followed by the development and refinement of the codebook. The authors held several sessions to refine and revise the codes. Once consensus was reached on the definitions of codes, themes, and sub-themes, the transcripts were imported into NVivo version 10, a qualitative analysis software package [47], which was used to manage the data and assist in the application of codes to the remaining transcripts. In applying coding, the authors read the transcripts to identify the themes occurring most frequently across transcripts and revised the emergent themes prior to the identification of the final themes and sub-themes. This approach enabled the researchers to identify, analyse and report patterns or themes in the data [46].

### Trustworthiness

The authors used a number of strategies to ensure trustworthiness. The lead author used reflexivity as a strategy to attain credibility by setting aside all preconceived ideas about the phenomenon under investigation as outlined in Gearing [48]. The lead author engaged in continuous peer debriefing with MM and SM as supervisors of the study in the form of frequent meetings through the project life. In addition, all the authors engaged with data analysis and interpretation. Other strategies to ensure credibility included; interviewing the participants in their own language, investigator triangulation, and transcribing the interviews verbatim to reflect the views of the participants. Lastly, the lead author kept an audit trail of the procedure and processes followed in the performance of the study [44].

### Ethics

The study received ethical clearance from the Sefako Makgatho Health Sciences University Research and Ethics Committee (SMUREC/H/68/2015: PG). Participation was voluntary and the participants provided written informed consent and all were 18 years of age or older. The authors used pseudonyms to report the data and maintained confidentiality at all times.

## Results

### Demographic characteristics of the participants

The ages of the 50 participants that were interviewed, ranged from 18 to 24 years with a mean age of 22 years (Standard deviation = 1 year). Most of them (68%) were female (n = 34). Half of them (n = 25) lived with grandparents in extended family households and 32% (n = 32) lived with siblings. Despite most of them living with adult family members in the household, almost a quarter 24% (n = 12) were responsible for their own care (Table 1).

**Table 1:** Sociodemographics of orphaned early school leavers (n = 50).

Characteristics		Number	Percentage
Age	18–20	17	34
	>21	33	66
*Mean age (SD)*	*22 (1)*		
Gender	Male	16	32
	Female	34	68
Who do they live with	Alone	5	10
	Siblings	16	32
	Grandparents	25	50
	Guardians	3	6
	Boyfriend	1	1
Person responsible for their care	Themselves	12	24
	Siblings	8	16
	Extended family	26	52
	Guardians	3	6
	Boyfriend	1	2
Number of people in the house	0–2	38	76
	>2	12	24

Table 2 presents data on the age, gender, and grade in which the participants left school. More (34) females than boys are early school leavers (34 vs. 16), and 43 out of 50 dropped out in high school. More than half (27) were between the ages of 15–18 years when they dropped out of school and 23 were aged above 18 years when they dropped out. Almost all (40) reported to have repeated at least one grade.

**Table 2:** School dropout profile of participants.

Variables	Age of leaving school		
	Total (n = 50) Freg./percent	15–18 (n = 27) Freg./percent	>18 (n = 23) Freg./percent
**Gender** `			
Female	34 (68)	19 (70)	15 (65)
Male	16 (32)	8 (30)	8 (35)
**Grade of dropping out**			
1-7^th^ grade	7 (14)	5 (18)	2 (9)
8-10^th^ grade	30 (60)	16 (59)	14 (61)
>10^th^ grade	13 (26)	6 (22)	7 (30)
**Level of dropping out**			
Primary level	7 (14)	5 (18)	2 (9)
High school	43 (86)	22 (81)	21 (91)
**Repeated any grades**			
Yes	40 (80)	18 (67)	22 (96)
No	10 (20)	9 (33)	1 (4)
**Number grades repeated**			
Once	21(52)	10 (56)	11 (50)
Twice	16 (40)	7 (39)	9 (41)
>3	3 (8)	1 (5)	2 (9)

### Findings from the interviews

Five themes describe the psychosocial effects of maternal death on the participants, namely; hunger and food insecurity, unequal treatment in the household, negative psychological emotions, prolonged bereavement, and maintaining silence.

### Hunger and food insecurity

The participants in this study lived in extended family households and in youth headed households with constrained resources since they were outside the threshold for social grant support. Poverty is a risk for lack of food as well as emotional and behavioural distress for orphans. The literature shows that orphans who are food insecure are at risk of developing emotional distress. The participants related how they used go to bed hungry, leave for school without breakfast, and come back to a home with no food. Hunger contributed to their leaving school before completing high school. They worry about food for themselves, their siblings, and even their own children because they continue to go to bed hungry.

*“I use to come back from school and find that there’s no food in the house and I could not study*. *I was a very bright learner during my school days*. *I used to go to school hungry without even a cup of tea*. *During lunch break*, *learners would go to their homes to eat*, *but I did not get anything at home*. *Sometimes I would go back to school but I would fail to concentrate*. *When the teacher teaches*, *you worry about what you will eat after school*. *I would not eat the whole day…*, *there was no bread after school*. *We would have no choice but wait till the evening meal”*

(Blessings, 22 year-old male).

“*My reason for dropping out was going to school without food*, *so I realised that it’s not good because still I wouldn’t even concentrate with an empty stomach*, *it’s painful*, *imagine you eat only at school…*., *then you think what you will eat again”*

*(Musa*, *21 year old female)*.

### Unequal treatment in the household

The data revealed that orphans experienced discrimination as compared to the kin children where they lived. Half of the participants (25) lived with grandparents in extended family households and 21 lived in youth headed households. The narratives revealed that their relatives treated them differently from their own children such as making hurtful remarks. Research suggests that ill treatment in extended households is the main reason orphans opt to live in youth headed households.

*“When my grandmother buys something she buys for XXX [her aunt’s] children*. *If she buys shoes for my aunt’s child*, *then I then have to go around the community to ask for help so that I can also buy shoes for my siblings*. *Sometimes when I complain and say I want this and that she says go and ask your mother*, *and I tell her my mother is dead and she would say go to the cemetery and ask her for that”*

(Mpho, 20 year-old female).

### Negative psychological emotions

Research has established that orphans suffer from psychological problems and exhibit high internalising problems. Their narratives reflected multiple long-term negative psychological emotions. They described internalising depressive symptoms such as hopelessness, self-isolation, sleepless nights, lack of peace, constant pain, and suicidal ideation.

#### Lack of peace

*“I was not at peace after my grandmother told me that I will never see my mother again*. *I never found peace after that*. *I couldn’t sleep at night because I kept thinking about my mother; sometimes I would wake up at night and go to my grandmother’s room to sleep there and when my grandmother wakes up at night she would find me awake and she would ask me why I couldn’t sleep*? *I told her that I couldn’t sleep without my mother”*

(Themba, 22 year-old male).

#### Self-isolation

“When I miss my mother I sit alone”

(Mpho, 20 year-old female).

“I stay the whole day in my room listening to music”

(Blessings, 22 year-old male).

#### Hopelessness

***“****After my mother passed away things changed and its difficult when you think of leaving home*, *where will I go because I cannot go and live at my friend’s home because they will get tired of me”*

(Mpho, 20 year-old female).

“I am considering becoming a driver because I think there’s nothing for me at the moment”

(Gift, 24 year-old male).

#### Constant emotional pain

*“Taking a deep breath… it’s painful… it’s very painful*. *We do not get used to that idea of not having a mother*, *for us not to have a mother changed us… our life style [participant crying] even our life has changed… it was never the same as before”*

(Thembi, 20 year-old female).

*“Sometimes I feel like crying when I look at my siblings*. *It is painful*. *I wish they were still here [the parents]”*

(Thandi, 22 year-old female).

#### Suicidal ideation

*“There was a time when I had some problems and I tried to kill myself*. *They found me before it was too late that’s when they took me to a social worker”*

(Thembi, 20 year-old female).

### Prolonged bereavement

The narratives revealed that the wounds inflicted by the death of the mother had not healed for a number of the participants in this study. Many had not had a chance to grieve for the loss of their mothers. They painfully remembered the death of the mother as if it happened yesterday. Yearning for the mother emerged from their narratives as a major sub-theme.

### Yearning for the mother

The participants had this strong belief that, had the mother not died, things would have been better and there would be someone there for them. The constant yearning for the mother triggered changes in their lives that they perceived were happening because of the mother’s death. They said that things would have been different if the mother had been around to show them love, to support them, to provide, to care, and to encourage them.

#### The mother’s love and understanding

*“I still needed my mother’s love*. *It is too difficult to be without a mother*. *There will never be a person who will understand you like your mother*, *who will listen to you and never judge you*. *When my mother was alive*, *she could see me when I had problems*. *Now there is no one who sees me if I am not okay*. *If my mother were still around*, *she would see me*. *She would then ask me and we will talk about it”*

(Gift 24 year-old male).

*“My mother’s place is always there at home*. *She is still needed…”*

(Thembi, 20 year-old female).

#### The mother’s support

*“Sometimes I think that if she was still alive I would have been able to continue with my education*. *She was going to encourage me”*

(Gift 24 year-old male).

“*It’s not nice*, *it’s painful*, *because you find that certain things you want to discuss it with a parent*, *as for your mother*, *certain things you want to discuss with your mother*, *but she is not there”*

(Lorato, 21 year-old female).

#### The mother’s moral compass

*“I think that if my mother was alive maybe things would have been better*, *because there are things that hurt me and I end up doing wrong things and feel bad about it the following day*. *For an example*, *I find a guy because I want money*, *I sleep with him*, *then the following morning I feel bad about it*. *That is why I say if my mother was alive such things would not be happening*. *Maybe everything that I wanted I was going to get it even if I would not get everything but some of it”*

(Nomthandazo, 20 year-old female).

#### Yearning for answers

*“Yoh… I ask myself*, *if mom was still alive*, *what drove her to abandoning me*?*”*

(Lucky, 21 year-old female).

*“Growing up without your parent is very difficult … you know … I really need my mother by my side*. *Like I am not right without her because there are many things … questions that I have to ask other people”*

(Lethabo, 21 year-old female).

### Maintaining silence

The narratives revealed that the adults and the participants used silence in dealing with the illness and death of their mothers. In many societies in SSA, the parents’ illness is often kept secret from the children during the illness and after the death, which is a source of emotional pain, resentment, anger, and unresolved grief.

#### Silence about parental illness

In response to questions about the illness and cause of death of their mothers, the narratives revealed that the issue of silence about the nature and cause of illness of their mothers was a source of concern for most of the participants. They told how the extended family members chose to be silent about the cause of illness and death of their mothers despite their direct involvement in the care of them in their terminal stages.

*“I nursed her but I didn’t know what she was suffering from*. *I only found out about her illness after her death*. *I found out when I was scolded and being told that my mother died of AIDS and I will also die of AIDS”*

(Gift, 24 year-old male).

“I didn’t know what was wrong with her until I got her death certificate written pneumonia but even today I still have questions of what type of pneumonia that it was”

(Lethabo, 21 year-old female).

### Silence about their feelings

The participants used silence to deal with the death of their mothers. They opted not to talk to others about things that bothered them and did not discuss their orphan status with others as a protective measure from rejection, stigma, and pain.

*“I don’t talk*. *If I am angry*, *I alone will know*. *If my mother was still around she would see me*, *she would then ask me and we would talk about it”*

(Gift, 24 year-old male).

“I bottle everything inside”

(Thandi, 22 year-old female).

#### Silence to avoid being labelled

“*I don’t want someone feeling pity for me because that brings back memories that I lost my parents while I was still young*, *so I don’t want someone who will say ‘Shame these are orphans’”*

(Angel, 23 year-old female).

## Discussion

The study aimed to explore how maternal death affects the psychosocial wellbeing of orphaned youth. We found that the participants lived in socially depressed environments characterised by absolute poverty [7, 19]. The only source of income for most of the households where they lived was the old age pension grants of their elderly relatives. All the participants were 18 years and older, which is the cut-off age for receiving child social grants in South Africa. This meant that they had no financial means of taking care of themselves except in rare instances where they performed menial casual jobs. Those who had assumed the role of taking care of their younger siblings relied on meagre social grants received on behalf of their siblings. The financial constraints observed in this study are consistent with those found in other studies [49–51]. Research has established the fact that poverty is a key cause of distress among orphans, likely leading to emotional and behavioural distress [51].

The study revealed that lack of traditional kin support increased material deprivation of the participants, as 21 orphans were household heads. Equally, those who stayed with extended families (25) lived with elderly relatives in intergenerational households. In such a household, the elders stay with the adults and young children and provide for their households by using their old-age social grants [52]. It is in these households that the participants were often subjected to unequal and unfair treatment, compared with other children in the household [35]. The unfair treatment included verbal abuse; for example, one orphan was told, to go and ask for the things that she needed from her mother in the graveyard. This kind of treatment is termed an “unsympathetic gesture” made by those who care for the orphans and is associated with increased emotional and social problems in orphans [53, 54]. The current study and others [55–58] found that such unequal treatment and gestures trigger emotional pain and feelings of being unloved. It is important that children are prepared for parental death so that they are less traumatised by the experience and are able to cope with it better [35].

The narratives revealed increased levels of psychological distress manifested in prolonged pain, despondency, hopelessness, anxiety and prolonged bereavement. It is an expectation in society that grieving individuals, including children, go through a number of stages such as feelings of shock, denial, sadness, anger, anxiety, guilt and, in the end, acceptance [59]. However, their narratives showed a preoccupation with the death of the mother, despite the length of the period of her death. They found it difficult to accept the death of the mother and often wondered how they were going to make it through life without her [51]. In the current study, this was reflected in repeated statements preceded by words such as “If my mother was alive…”

In addition, they feared for their future and experienced a lack of peace long after the death of the mother. A similar observation was reported by Runhare and Gordon [28] who stated that orphans experience hidden grief, which manifested in recollections about their late parents, deep sadness and a lack of peace. Likewise, fear and lack of peace were among the negative emotions experienced by orphans in studies conducted in Zimbabwe [60, 61].

The narratives revealed that the participants used silence as a coping strategy although it is a negative coping mechanism. They spoke of bottling up their feelings when it comes to matters relating to the death of the mother. It is worth noting that they are from the same households and society that are characterised by a culture of silence, and the adoption of this behaviour of silence is explained as children mimicking the behaviour of the adults around them [62]. Nevertheless, research shows that denying children an opportunity to express their emotions results in negative manifestation of feelings characterised by anger, frustration and anxiety [35, 63]. The participants in the current study expressed the same emotions. The findings suggest that it is important to provide psychological services for orphans immediately following the death of a parent. However, such services should be culturally appropriate and be provided to extended family members in the households.

Food insecurity was one of the major problems experienced by the participants, and hunger played a big role in their lives. They were repeatedly subjected to hunger at home and sometimes had to go to bed hungry. This finding correlates with that in the literature, which reports that orphans frequently go to bed hungry, which places them at risk of mental distress [64]. Hunger was a source of pain and sadness for them, and their narratives revealed that having to go to school on an empty stomach and experiencing frequent hunger drove some of them to leave school early. They told how hunger affected their concentration at school, which resulted in poor school performance. This is consistent with other studies in SSA which highlighted that hunger affected the educational performance of orphans negatively [7, 22]. Most of the participants (40 of the 50) had repeated at least one grade, whereas nineteen had repeated more than once. It should be noted that the hunger that force them out of school continued after they left school, more so because they remained unemployed.

Early school leaving is associated with psychological distress among orphans [26, 51]. The study found that leaving school early induced emotional distress for the participants. Their narratives showed that they worry about not being in school and felt that there was no hope in their future because they had dropped out of school. There is evidence that education increases children’s survival and development prospects [65]. Therefore, early school leaving has negative implications for future economic development as it reduces employment opportunities. Of public health concern is that education is one of the strongest predictors of long-term health and health services utilization. This suggest that reduced number of years in schooling increases levels of risky health behaviours [66, 67]. In the context of HIV/AIDS, the school may be the one place where children can obtain accurate information about HIV prevention, as well as life skills that empower them to resist unwanted sex and early marriage [65]. Without valuable life-skills, out-of-school orphans are more likely to face social, psychological, economic and health problems as they grow up [68].

### Limitations

The study had limitations in that it was limited to one rural municipality, and the findings can therefore not be generalised to other parts of the country and to orphans in urban areas. In addition, the findings are based on a small sample of orphaned youth and cannot represent the psychosocial wellbeing of orphans who left school before completion. The findings may also be limited as they are based on the narratives of the participants only, and the study did not use standardised tools to measure psychosocial wellbeing. A larger sample using a quantitative survey and assessing mental health of orphans in and out of school using a standardised tool is required. One other limitation of the study is that we did not collect data on the HIV status of the orphans and could not report on own HIV status as a source of stress. Nevertheless, the focus of the study was on how maternal death affects the psychosocial wellbeing of orphans who dropped out of school prematurely.

## Conclusion

Maternal death has a negative impact on the psychosocial wellbeing of the participants even after they crossed the 18 years threshold of orphan hood resulting in the development of internalising depressive symptoms. They suffer from emotional distress and prolonged bereavement characterised by perpetual yearning for their mothers which negatively affected their ability to develop coping strategies. They resorted to silence as the only coping strategy, and that further exacerbated their emotional distress and led to isolation, sadness, hopelessness, fear and lack of peace.

Dropping out of school was also a major stressor in their lives and contributed to their poor psychosocial wellbeing. Moreover, the state of their living conditions was overwhelming and they foresaw no opportunity for them to complete school.

The study found that in this setting, the extended family still plays a role in the care of orphans in spite of the socially depressed environment where most of the households belong. Therefore, it is important to consider this when interventions are developed to address the needs of orphans.

It is important that the relevant policies should include the continuation of support for those over the age of the 18 years threshold who experience maternal death–the same as for children or adolescents. In light of the withdrawal of the social grant system for this group of orphans, NGOs and community networks should play a key role in material and emotional support in a sustainable manner through government funding. There is need for interventions that will improve their skills to prepare them for the job market or self-employment. This could be achieved through the Technical and Vocational Education and Training Colleges.

Although the study population consisted of early school leavers, the proposed intervention should also focus on orphans who are still in school to mitigate the risk of leaving school early before completing high school. The school is in a position to mitigate against issues like poor academic performance, high levels of psychosocial distress, and early school leaving among orphans.

The lack of routine screening for mental health of orphans in South Africa increases their vulnerability to undiagnosed depression. While this is not the competency of the school, the school health services presently being offered in South Africa have a critical role to play in the development of interventions for the mental health screening of orphans in schools. It is crucial that the school health services further provide counselling services to assist learners to deal with problems that affect their educational performance like the death of a parent and other related psychosocial emergencies.

Schools can also play a part in providing for this support by empowering the teachers with skills to identify orphans who are at risk and refer to appropriate service provides such as social workers, psychologists, and other social services. There is substantive evidence that teacher are more likely to support orphans when they know about their living circumstances. One of the key challenges of orphans is lack of support in getting their homework done; the school could provide space, resources, and support in this regard.
